# TGF-β Enhances Immunosuppression of Myeloid-Derived Suppressor Cells to Induce Transplant Immune Tolerance Through Affecting Arg-1 Expression

**DOI:** 10.3389/fimmu.2022.919674

**Published:** 2022-07-07

**Authors:** Peng Cao, Zejia Sun, Feilong Zhang, Jiandong Zhang, Xiang Zheng, Baozhong Yu, Yong Zhao, Wei Wang, Wei Wang

**Affiliations:** ^1^ Department of Urology, Beijing Chaoyang Hospital, Capital Medical University, Beijing, China; ^2^ State Key Laboratory of Membrane Biology, Institute of Zoology, Chinese Academy of Sciences, Beijing, China

**Keywords:** myeloid derived suppressor cells (MDSCs), TGF-β, ARG-1, transplant, Immune rejection, immune tolerance

## Abstract

Myeloid-derived suppressor cells (MDSCs) are a class of heterogeneous myeloid cells, which play an important role in immunosuppression. We intended to find an effective method that can produce MDSCs with significantly better efficiency and promote immune tolerance for transplant rejection through cell therapy. It has been reported that granulocyte and macrophage colony-stimulating factor (GM-CSF) could induce MDSCs *in vitro* to cause immunosuppression. In the present study, transforming growth factor β (TGF-β) was added to the induction system, and flow cytometry analysis was used to detect the phenotypes of induced MDSCs. Their potential immunosuppressive function and mechanisms were determined by co-culturing MDSCs with stimulated T cells *in vitro* and transferring MDSCs to the skin grafted C57BL/6J mouse models *in vivo*. It was found that the addition of TGF-β could effectively cause bone marrow cells to differentiate into a group of cells with stronger immunosuppressive functions, thereby inhibiting the proliferation of stimulated T cells. The population of CD11b^+^Gr-1^+^ MDSCs also increased significantly as compared with GM-CSF alone treatment. While detecting for immunosuppressive effectors, we found that expression of arginase 1 (Arg-1) was significantly upregulated in these MDSCs, and inhibitor of Arg-1 significantly suppressed their immunosuppressive capabilities. Moreover, an adoptive transfer of these cells significantly prolonged survival of allo-skin and improved immune tolerance *in vivo*. These findings indicated that TGF-β + GM-CSF could serve as an effective and feasible method to induce powerful immunosuppressive MDSCs *in vitro*. Thus, TGF-β + GM-CSF–induced MDSCs may have a promising role in prevention of the graft rejection.

## Introduction

The bone marrow cells can develop and differentiate into macrophages, dendritic cells, and neutrophils under normal physiological conditions and enter corresponding tissues and organs to exert their specific immune functions (1). However, under various pathological conditions, such as tumors, trauma, autoimmune diseases, burns, and transplantation, the bone marrow cells can, in turn, differentiate into a group of heterogeneous cell population that can exhibit powerful negative immunomodulatory effects and have been named myeloid-derived suppressor cells (MDSCs) (2).

MDSCs are usually found in the peripheral blood, lymph organs, and bone marrow. MDSCs can effectively gather in large numbers around the tumor tissues and inflammatory sites (3). The characteristic phenotype of MDSCs has not been confirmed yet. However, in the mice, MDSCs have been defined as a group of CD11b^+^ Gr-1^+^ cells (4). Further studies have found that there are two different subtypes of MDSCs, namely, granulocytic myeloid suppressor cells (G-MDSCs) with CD11b^+^Ly6G^+^Ly6C^−^ and monocytic myeloid suppressor cells (M-MDSCs) with CD11b^+^Ly6G^−^Ly6C^+^ (5). In humans, the recognized phenotype of MDSCs is CD11b^+^CD33^+^HLA-DR^−^, whereas the phenotypes of M-MDSCs and G-MDSCs are CD11b^+^CD33^+^CD14^-^CD15^+^ and CD11b^+^CD33^+^CD14^+^CD66b^+^, respectively. In addition, it has been previously reported that there existed another group of immature MDSCs, which can express Lin^−^HLA-DR-CD33^−^, and are located in the peripheral blood, which are defined as early-stage MDSC (eMDSCs) (6). However, with increase in reported research findings related to MDSCs, questions about MDSCs have also gradually emerged. Some studies have shown that the phenotype of MDSCs may not be invariable, and the subtypes have mutual transformation and even can develop into the mature immune cells with a possible change of the surrounding environment (7, 8). It has been also found that a variety of cytokines can potentially mobilize the expansion and activation of MDSCs. The factors that can promote MDSCs expansion include cyclooxygenase 2 (Cox 2), prostaglandin, stem cell factor, colony-stimulating factors (CSFs), vascular endothelial growth factor (VEGF), and interleukin 6 (IL-6). Most of these factors participate in the regulation of cell survival, proliferation, differentiation, and apoptosis through the modulation of the Janus kinase/signal transducer and activator of transcription (JAK/STAT) signaling pathway (9). Moreover, the immunosuppressive functions of MDSCs also require the induction of several cytokines, including interferon-γ (IFN-γ), IL-13, IL-4, Toll-like receptor (TLR) ligands, and transforming growth factor β (TGF-β). These cytokines can mediate the function of MDSCs through activating STAT6, STAT1, and nuclear factor kappa-B (NF-κB) signal transduction pathways (10–12). The primary difference between MDSCs and other myeloid-derived cells is that they can exert immunosuppressive effects, and their main inhibitory target is T cells. MDSCs can also regulate expression of several important inhibitory effectors, such as inducible nitric oxide synthase (iNOS), arginase 1 (Arg-1), heme oxygenase 1 (HO-1), cyclooxygenase-2 (Cox2), indoleamine 2,3-dioxygenase (IDO), TGF-β, IL-10, and L-selectin. This can lead to a significant inhibition of the immune response of T cells, inducing T-cell apoptosis and can promote an expansion of regulatory T cells (Tregs), thereby actively participating in the establishment of robust immune tolerance (13).

Colony-stimulating factors (CSFs) can function as indispensable stimulating factors for regulating hematopoiesis. They can facilitate the proliferation and differentiation of the hematopoietic stem cells at different developmental stages and can be divided into different categories based on the characteristics, such as granulocyte and macrophage CSF (GM-CSF), granulocyte CSF (G-CSF), macrophage CSF (M-CSF), and multi-CSF (also known as IL-3) (14). A few studies have reported that CSFs alone or in combination with other bioactive molecules can be used to induce MDSCs *in vitro* (15). For example, Rossner et al. found that GM-CSF could stimulate the bone marrow cells to differentiate into a group of CD11b^+^Ly6C^+^Ly6G^low^ CD11c^−^CD31^+^ cells *in vitro*, which could suppress immune response that triggered by allogeneic antigens (16). However, there is no unified protocol established so far for induction of MDSCs *in vitro*. In our previous study, we successfully induced immunosuppressive MDSC *in vitro* by combining relevant inflammatory factors with CSFs (17–19). We wanted to find a cytokine combined with CSF that could more easily and efficiently induce MDSCs with immunosuppressive functions. TGF-β is widely present in the organ tissues of many organisms and is a large class of regulatory cytokines that can promote cell proliferation and directed differentiation in the body. It has been found that TGF-β has immunomodulatory functions (20), mainly in regulating the activation, proliferation, and function of immune cells to induce immune tolerance and maintain self-homeostasis. It has been shown that it can induce Foxp3 to increase Tregs (21). The present study intends to add TGF-β into the induction system with GM-CSF to construct a feasible and efficient induction protocol and explore the possible mechanisms of its immunosuppressive function. We also aim to find a promising cell therapy with MDSCs to promote immune tolerance to transplantation procedure.

## Materials and Methods

### Experimental Animals

C57BL/6 (B6) mice used in this study, aged 6–8 weeks, were all purchased from Spelford Laboratory Animal Company (Beijing, China). All the mice were specific pathogen–free (SPF) animals and housed and bred in an SPF facility in the Institute of Zoology, Chinese Academy of Sciences. All the Animal experiments were approved by the Ethics Committee of Beijing Chaoyang Hospital (Beijing, China) and conducted in strict compliance with International Regulations on Management of Experimental Animals.

### Reagents and Antibodies

Recombinant mouse GM-CSF and human TGF-β were purchased from PeproTech (Rocky Hill, NJ, USA). Flow cytometry (FCM) antibodies (Abs) including anti-mouse (anti-m) CD11b-FITC, anti-mGr1-PE, anti-mLy6C-FITC, anti-mLy6G-PE, anti-mF4/80-PE-Cy5, anti-mCD11c-PE, anti-mCD80-FITC, anti-mCD86-FITC, anti-mI-Ab-FITC, anti-mCD31-PE, anti-CD40-PE, anti-mCD62L-PE, anti-mCD115-PE, anti-mCD124-PE, anti-mCD274-PE, and anti-mFoxp3-PE were purchased from eBioscience (San Diego, CA, USA). Anti-mIgG1-FITC, anti-mIgG2a-FITC, anti-mIgG2b-FITC, anti-mCD4-PE, anti-mCD8-PE-Cy5, and anti-mCD25-PE-Cy5 were purchased from BD Biosciences Pharmingen (San Diego, CA, USA). Anti-mIFN-γ-PE, anti-mTNF-α-PE, anti-miNOS-PE, anti-mArg-1-PE, anti-mTGF-β-PE, and anti-mIL-2-PE were purchased from Thermo Fisher Scientific (Waltham, MA, USA).

### Induction of MDSCs *In Vitro*

Bone marrow cells were flushed from the femur, tibia, and ilium of the B6 mouse with a syringe containing RPMI 1640 medium. ACK lysis buffer was used to lyse the erythrocytes. The leftover cells were seeded in 10-cm dishes (Corning, USA) for 2 h. Afterward, the non-adherent cells that can serve as progenitor cells for induction were collected. Non-adherent bone marrow cells (2 × 10^6^) were seeded in 6-cm dish with GM-CSF (40 ng ml^−1^) and TGF-β (2 ng ml^−1^) in 4 ml of complete medium (CM) [RPMI 1640 medium supplemented with 2 mM L-glutamine, 10 mM HEPES, 20 μM 2-mercaptoethanol, penicillin (150 U ml^−1^), streptomycin (200 U ml^−1^), and 10% heat-inactivated FBS] for 4 days in an incubator at 37°C and 5% CO_2_. Afterward, we collected all the cultured cells for further experiments without any purifying to minimize cell damage and loss. GM-CSF alone–treated cells were used as a control.

### Assays to Monitor the Suppression of Activated T Cells

Induced MDSCs were collected on the fourth day in the complete culture medium and adjusted to a concentration of 2×10^6^ ml^−1^. Diluted MDSCs (100 μl) were added to 96-well flat bottom plates thereafter. Meanwhile, T-cell proliferation assay was described as in the previous study (17). Carboxyfluorescein diacetate succinimidyl ester (CFSE)–labeled splenic lymphocytes (2×10^6^ ml^−1^) were stimulated by 4 μg ml^−1^ of ConA to induce the proliferation, and then, 100 μl of the splenocytes were added to the wells containing MDSCs and cultured at 37°C, 5% CO_2_ for 3 days. FCM analysis was used to detect the dilution ratio of CFSE in CD4^+^ and CD8^+^ T cells. PMA (50 ng ml^−1^; Sigma-Aldrich), ionomycin (500 ng/ml; Sigma-Aldrich, St. Louis, CA, USA), and GolgiStop (BD Pharmingen, San Diego, CA, USA) were added to the 96-wells 6 h before harvesting the cells to stimulate these co-cultured cells. FCM analysis was used to detect the production of inflammatory cytokines by effector T cells. iNOS inhibitor NG-monomethyl-L-arginine (L-NMMA; Sigma-Aldrich), arginase inhibitor Nω-hydroxy-nor-L-arginine (nor-NOHA; Calbiochem, Burlington, NJ, USA), and L-Arginine (Sigma-Aldrich) were used at the beginning of the co-culture experiment to test the potential effect of inhibitory effector molecules on the proliferation of the stimulated T cells.

### Flow Cytometry Assay

The samples in each tube of 100 μl of PBSA (phosphate-buffered saline, PBS with 0.1% bovine serum albumin, BSA) containing 2–5 × 10^5^ cells under test were incubated with appropriate fluorescent Abs for 30 min at 4°C in the dark for surface staining. Thereafter, the samples were washed using 3 ml of PBSA and centrifuged at 1,500 rpm and 4°C for 5 min to remove the free Abs in the supernatant. The stained cells were resuspended with 300 μl of PBSA for analyzing the various surface marker. Intracellular staining was usually performed after staining the cell surface. Cytofix/Cyoperm solution (BD Pharmingen) was used for the fixation and membrane permeabilization of the collected cells in the sample. Then, intracellular staining was performed according to the method described above for surface staining. All the samples were tested on a Beckman Coulter Gallios Flow Cytometer (Beckman Coulter, Brea, CA, USA), and forward scatter (FCS) express software (*De Novo* Software, Los Angeles, CA, USA) was used to analyze the collected data. Live cells were gated by side and forward scatter. Then, the percentage of the objective cells is determined by the ratio of stained cells to live cells. A minimum of 30,000 events were obtained per sample.

### Real-Time Quantitative PCR Analysis

MicroElute Total RNA Kit (Omega Bio-tek, Doraville, GA, USA) was used to extract total RNA from induced MDSCs, and reverse transcription was performed according to the manufacturer’s instructions for moloney murine leukemia virus (M-MLV) superscript reverse transcriptase (Takara Bio, Shiga, Japan). Real-time quantitative PCR (qPCR) was performed by using the CFX96 Real-Time System (Bio-Rad Laboratories, Hercules, CA, USA), and relative changes in mRNA expression were normalized to that of hypoxanthine phosphoribosyltransferase (HPRT) using the 2^−ΔΔCt^ method. The primer sequences of different genes used in the present study are shown in **Supplementary Table 1**.

### Detection of Nitric Oxide in Co-Culture Medium

Nitric oxide (NO) assay kit was purchased from Jiancheng bioengineering institute (Nanjing, China). Reagent I and reagent II (200 μl) were mixed with 100 μl of supernatant of co-culture medium for 10 min. Thereafter, 160 μl of supernatant was taken as a sample after centrifugation at 3,500 rpm for 15 min. The samples and chromogenic agent were then added to the detection wells for 15 min according to the manufactory’s instructions. The absorbance optical density (OD) value was measured using a microplate reader (Biotech, Los Angeles, CA, USA) at 550 nm. The concentrations of NO in samples were determined by a standard curve generated using serial dilutions of sodium nitrite.

### Arginase Activity Assay

An arginase activity assay kit, which was purchased from Abcam (Cambridge, Cambs, UK), was used to detect the arginase activity of the cells in the co-culture medium. The standard solutions and experimental samples were prepared according to the manufactory’s instructions. The substrate mixture was then mixed with the samples and incubated at 37°C for 20 min. After addition of the reaction mix to the reaction system, the absorbance for all the samples was measured immediately on a microplate reader at 570 nm. Arginase activity was thereafter calculated on the basis of a standard curve.

### Mouse Skin Transplantation

The tail skin of male B6 mice was prepared as a graft to be transplanted to the dorsal part of the female B6 recipients. Induced MDSCs at a density of 5 × 10^6^ were adoptively transferred to the recipients by intravenous injection (i.v.) day 1 before surgery and day 7 after surgery respectively. The transplantation mice in another group was treated with MDSCs in combination with Arg-1 inhibitor nor-NOHA (40 mg kg^−1^) for 3 days by intraperitoneal injection. The condition of the skin grafts was observed and photographed every day after removing the bandage. The transplanted skin with redness, swelling, inflammation, and necrosis was considered to have a rejection. Thereafter, end point was determined when the rejection area was more than 90% of the graft.

### Hematoxylin-Eosin Staining

The grafted skin was acquired from transplanted mouse on the day 21 after operation and was fixed by in 10% buffered formalin overnight at room temperature. The skin were embedded in paraffin the next day. Then, skin tissue slices were deparaffinized in xylene (three times, 5 min each), rehydrated (100%, 90%, 80%, and 70% alcohol, 5 min each), dehydrated, and deparaffinized. Histological changes were detected by staining 5-μm-thick sections with hematoxylin-eosin (HE) staining. Images were acquired microscopically using a B 40 upright light microscope (Olympus, Tokyo, Japan).

### Donor-Specific Antibody Assay

The venous blood from the tail of the transplanted mice was obtained on days 7, 14, and 21 after the surgery, respectively. The blood serum in a quantity of 10 µl was mixed with 90 µl of splenocytes without the presence of erythrocytes of B6 male mice. The mixed cells were co-cultured at 4°C for 30 min. The residual serum in the mixture was then washed with PBSA solution. Thereafter, the cells were resuspended with 60 μl of PBSA solution. The levels of IgG1, IgG2a, and IgG2b Abs on the spleen cells were detected using FCM assay as described above.

### Statistical Analysis

All data have been shown as the mean ± standard error of mean (SEM). A two-tailed unpaired Student’s *t-* test was used for comparison between the two different groups. One-way ANOVA analysis was used for comparison among the multiple groups. The skin graft survival curves were calculated using the Kaplan–Meier method, and the log-rank test was used for graft survival comparison. GraphPad Prism software version 11 (GraphPad Software, La Jolla, CA, USA) was used for statistical analysis. A *P-*value < 0.05 was considered to be statistically significant.

## Results

### Addition of TGF-β Facilitated Progenitor Cells From the Bone Marrow to Differentiate Into MDSCs

**Figure 1:**
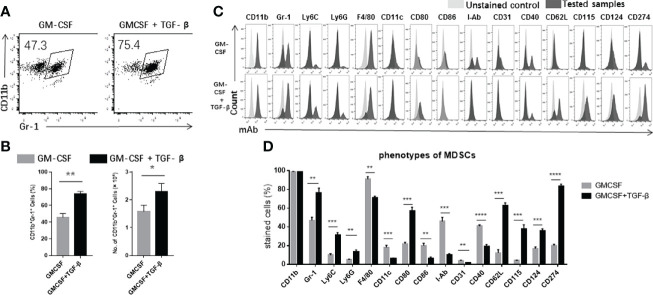
The characteristics of TGF-β + GM-CSF–induced MDSCs. **(A)** The precursor cells from the bone marrow cells were cultured in GM-CSF alone or TGF-β + GM-CSF as described in Materials and Methods for 4 days. The percentages of CD11b^+^Gr-1^+^ MDSCs have been shown by FCM analysis. **(B)** The proportions and numbers of CD11b^+^Gr-1^+^ MDSCs that were induced by either GM-CSF alone or TGF-β + GM-CSF. **(C)** FCM analysis showed the typical phenotypes of the cells stained by FITC-, PE-, PE-Cy5–, or APC-labeled anti-mouse-CD11b, Gr-1, Ly6C, Ly6G, F4/80, CD11c, CD80, CD86, I-Ab, CD31, CD40, CD62L, CD115, CD124, and CD274 Abs. **(D)** The proportions of the indicated markers have been summarized. The data have been shown as mean ± SEM (n = 3) and were analyzed by an unpaired two-tailed Student’s t-test, which were collected from three independent experiments. *P <* 0.05 was considered as statistically significant between the groups. *P < 0.05, **P < 0.01, and ***P < 0.001 upon comparison between the two groups.

First, we intended to characterize the features of TGF-β + GM-CSF as potential inducing factors to promote the differentiation of the precursor cells from the bone marrow into MDSCs. We used GM-CSF–induced MDSCs as a control group. However, upon comparison of the two groups of induced cells, we found that TGF-β combined with GM-CSF could significantly promote the precursor cells to differentiate into a larger proportion of CD11b^+^Gr-1^+^ MDSCs (**Figures 1A, B**). The number of TGF-β + GM-CSF–induced MDSCs was also significantly higher as compared with that in the GM-CSF alone induction group (**Figure 1B**). In addition, the bone marrow cells were cultured in the CM with or without TGF-β alone for 4 days. The proportion and numbers of CD11b^+^Gr-1^+^ MDSCs in the two groups were significantly lower than that in the GM-CSF–induced group or the TGF-β + GM-CSF–induced group (**Supplementary Figure 1**). Meanwhile, we examined the induced cells by Wright–Giemsa staining. We found that M-MDSCs and G-MDSCs in both of the inductive systems had no significant different in morphology. Most of MDSCs induced by TGF-β + GM-CSF were M-MDSCs (**Supplementary Figure 2**). We further explored the phenotypes of the two groups of induced cells, including CD11b, Gr-1, Ly6C, Ly6G, F4/80, CD11c, CD80, CD86, MHC class II molecules (I-Ab), CD31, CD40, CD62L, CD115, CD124, and CD274 (**Figures 1C, D**). Interestingly, the addition of TGF-β induced a significant increase in the expression of Gr-1, which is a characteristic molecule of MDSCs of mouse. Gr-1 can be further divided into Ly6C and Ly6G. We observed that Ly6C on the TGF-β + GM-CSF–induced cells increased more significantly as compared with Ly6G, which might indicate that TGF-β promoted the differentiation of myeloid cells into greater number of CD11b^+^Ly6C^+^ M-MDSCs. However, the expression level of F4/80, a unique marker of macrophages, was noted to be reduced in the TGF-β + GM-CSF–induced cells group. The expression of two different markers related to the dendritic cells, CD11c and I-Ab, was also observed to be decreased. The levels of co-stimulatory molecules, such as CD86 and CD40, expressed on the cells also decreased. However, another co-stimulatory molecule, CD80, increased in the cells that were induced by the treatment of TGF-β + GMCSF. Moreover, the expression of CD115 and CD124 related to the regulation of inhibitory functions of MDSCs also increased significantly. The expression level of CD274 on TGF-β + GM-CSF–induced cells also significantly increased but that of CD31 decreased markedly. The above results suggested that the addition of TGF-β to the induction system could effectively promote the bone marrow cells to differentiate into more immunosuppressive cells.

### MDSCs Induced by GM-CSF + TGF-β Increased Its Capability to Inhibit T-Cell Proliferation

**Figure 2:**
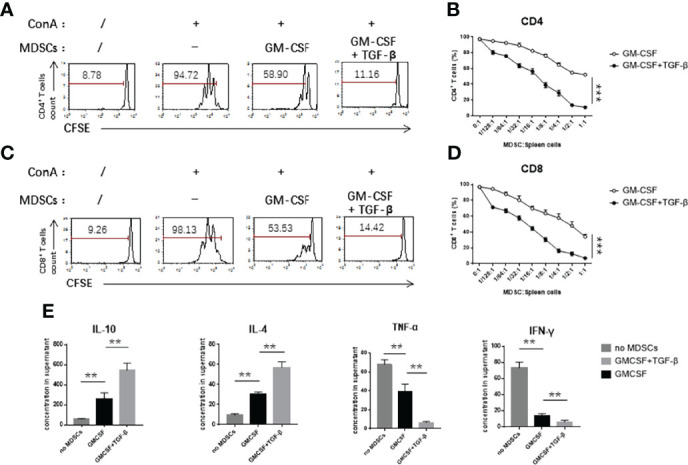
TGF-β + GM-CSF–induced MDSCs exhibited significant immunosuppressive functions. GM-CSF and TGF-β + GM-CSF–induced MDSCs were added to the stimulated T cells proliferation system at different concentrations for 3 days. **(A)** Typical samples of FCM analysis for T-cell proliferation of CD4^+^ (T cells: MDSCs = 1:1/2). **(B)** Percentage of CD4^+^ T-cell proliferation was measured by CFSE dye dilution co-cultured with induced MDSCs at indicated ratios. **(C)** Typical samples of FCM analysis for T-cell proliferation of CD8^+^ (T cells: MDSCs = 1:1/2). **(D)** Percentage of CD4^+^ T-cell proliferation was measured by CFSE dye dilution co-cultured with induced MDSCs at indicated ratios. **(E)** The levels of inflammatory cytokines, IL-10, IL-4, TNF-α, and IFN-γ in the co-culture supernatant were detected by ELISA assay (T cells: MDSCs = 1:1/2). Data have been shown as mean ± SEM (n = 3) and were analyzed by an unpaired two-tailed Student’s t-test and one-way ANOVA analysis, which were collected from three independent experiments. *P < 0.05, **P < 0.01, and ***P < 0.001 upon comparison between the two groups.

**Figure 3:**
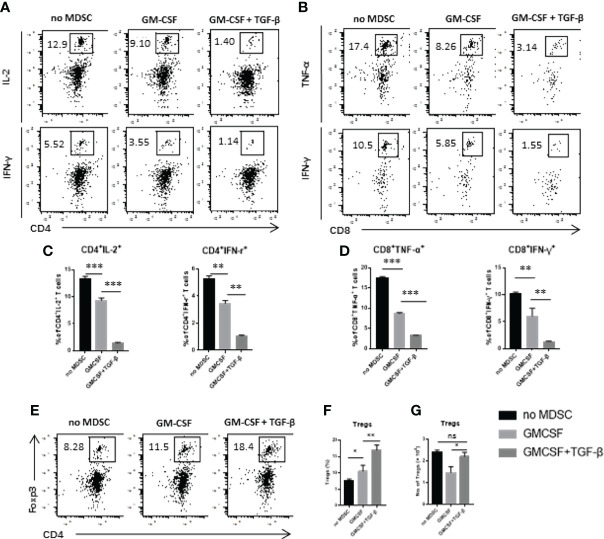
TGF-β + GM-CSF–induced MDSCs can effectively suppress cytokine production in the stimulated T cells and cause an increase in the number of Tregs. GM-CSF–induced and TGF-β + GM-CSF–induced MDSCs co-cultured with the stimulated T cell (stimulated cell–to–MDSC ratio = 1:1/2) for 3 days followed by adding PMA and ionomycin for stimulation. Typical samples of FCM analysis conducted for levels of the cytokine produced by the T cells in **(A)** and **(B)**. The percentages of CD4^+^and CD8^+^ T cells producing IL-2 and IFN-γ **(C)** and TNF-α and IFN-γ **(D)**, respectively, with or without induced MDSCs in the culture system have been summarized. **(E)** Typical samples of FCM analysis for CD4^+^Foxp3^+^ Tregs in the culture system with or without the induced MDSCs. **(F, G)** The percentages and numbers of Tregs in the co-culture have been summarized. The data have been shown as mean ± SEM (n = 3) and were analyzed by one-way ANOVA analysis, which were collected from three independent experiments. *P < 0.05, **P < 0.01, and ***P < 0.001 upon comparison between the different groups. ns, no significance.

We further investigated the inhibitory ability of induced MDSCs *in vitro*. We used concanavalin A (ConA) as a potential stimulatory factor to trigger the activation and proliferation of splenic T cells. After three days of co-cultivation with the different ratios of induced MDSCs, FCM assay was used to detect the dilution ratio of CFSE-labeled CD4^+^ (**Figures 2A, B**) and CD8^+^ T cells (**Figures 2C, D**). According to the results, it was found that, with an increase of the concentration of induced cells, the potential effect on inhibition of T-cell proliferation was significantly stronger. The cells induced by TGF-β in combination with GM-CSF exerted a stronger inhibitory effect on the T-cell proliferation as compared with the cells induced by GM-CSF alone. We used enzyme linked immunosorbent assay (ELISA) assay kits to test the expression levels of pro-inflammatory factors in the co-culture supernatant. We found that the concentration of pro-inflammatory cytokines, including TNF-α and IFN-γ, in the supernatant obtained from TGF-β + GM-CSF–induced MDSCs co-culture system was lower than as compared with the control group. However, the concentration of anti-inflammatory cytokines, such as IL-10 and IL-4, was significantly lower in the control group (**Figure 2E**). We further used FCM analysis to detect the activation of effector T cells and found that the inherent ability of CD4^+^ and CD8^+^ T cells to produce cytokines, such as TNF-α, IFN-γ, and IL-2, decreased more significantly in the TGF-β + GM-CSF–induced MDSCs group than that in the control group (**Figures 3A–D**). In the co-culture system containing TGF-β + GM-CSF–induced MDSCs, although Tregs could be found on day 0 in the T cells, the proportion of CD4^+^Foxp3^+^ regulatory T cells (Tregs) increased significantly (**Figures 3E, F**). The number of Tregs could sustain without significant decline (**Figure 3G**). In addition, we found that TGF-β + GM-CSF–induced MDSCs could only promote the Tregs with activating potential. The Tregs remained low level in the co-culture after the T cells were treated with CD25 Abs (**Supplementary Figure 3**). These findings thus possibly indicated that the induced MDSCs not only inhibited the activation and expansion of stimulated T cells directly but also participated in immune regulation by promoting the increase in the population of Tregs indirectly. Interestingly, the MDSCs that were induced by TGF-β + GM-CSF exposure could further promote these effects significantly.

### TGF-β + GM-CSF–Induced MDSCs Exerted Significant Immunosuppressive Effects Through the Arg-1

**Figure 4:**
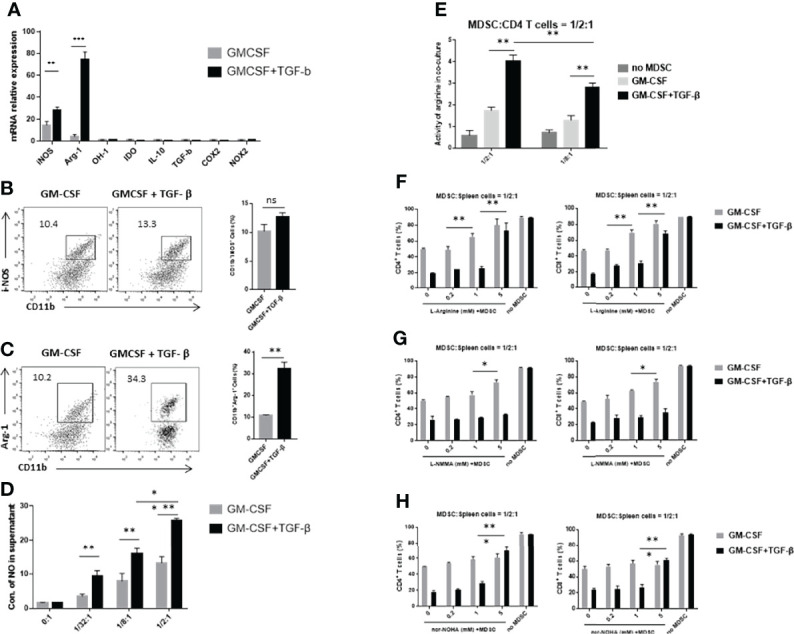
TGF-β + GM-CSF–induced MDSCs exert immunosuppressive functions through the Arg-1 pathway. **(A)** The mRNA expression levels of iNOS, Arg-1, OH-1, IDO, IL-10, TGF-β, COX2, and NOX2 in GM-CSF and TGF-β + GM-CSF–induced MDSCs were determined by real-time qPCR assay. Typical dot plot of FCM analysis conducted for levels of iNOS **(B)** and Arg-1 **(C)** produced by the induced MDSCs. The percentages of CD11b^+^iNOS^+^ and CD11b^+^Arg-1^+^ cells in the GM-CSF– and TGF-β + GM-CSF–induced system have been summarized. **(D)** GM-CSF– and TGF-β + GM-CSF–induced MDSCs in different concentrations co-cultured with stimulated T cells and the NO levels in the culture medium were detected. **(E)** The activity of arginine in the co-culture system (MDSC–to–stimulated cell ratio = 1/2:1 and 1/8:1) was detected. Administration of L-arginine **(F)**, iNOS inhibitor L-NMMA **(G)**, and Arg-1 inhibitor nor-NOHA **(H)**, respectively, in co-culture system to analyze the potential inhibitory effects of GM-CSF+TGF-β + GM-CSF–induced MDSCs on the stimulated T-cell proliferation (MDSC–to–stimulated cell ratio = 1/2:1). The data have been shown as mean ± SEM (n = 3) and were analyzed by an unpaired two-tailed Student’s t-test, which were collected from three independent experiments. *P < 0.05, **P < 0.01, and ***P < 0.001 compared between the different groups.

A number of previous studies have reported that MDSCs can produce different inhibitory effector molecules to exert their immunosuppressive effects. We used real-time qPCR to detect the mRNA expression levels of different inhibitory effector molecules, including iNOS, Arg-1, OH-1, IDO, IL-10, TGF-β, COX-2, and NOX2. We found that TGF-β + GM-CSF–induced MDSCs displayed an upregulated mRNA expression levels of iNOS and Arg-1 (**Figure 4A**). Then, we performed FCM analysis to compare the percentages of CD11b^+^Arg-1^+^ and CD11b^+^iNOS^+^ cells in the GM-CSF alone group and the GM-CSF + TGF-β group. There was no statistical significance between the expression level of iNOS in GMCSF-induced MDSCs and GMCSF + TGF-β–induced MDSCs (**Figure 4B**). However, the results showed that the ratio of CD11b^+^Arg-1^+^ cells in the GM-CSF + TGF-β group is more significant than that in the GM-CS alone group, which was consistent with the result of mRNA expression (**Figure 4C**). It has been reported that L-arginine could be possibly involved in the proliferation of T cells. As a substrate, it can be effectively metabolized by iNOS and Arg-1 to produce NO, citrulline and urea ornithine, respectively (22). Therefore, upon the detection of the concentration of NO and arginase activity in the co-culture system, it was found that the levels of both of them in the TGF-β–induced MDSCs group were significantly higher as compared with the control groups (**Figures 4D, E**). After supplementing L-arginine in the co-culture system, it was observed that the proliferation of T cells was substantially restored. However, upon comparison with the GM-CSF alone–induced MDSCs group, in which the proliferation of T cells increased progressively with an increase of L-arginine concentration, the TGF-β + GM-CSF–induced MDSCs group could only restore T-cell proliferation upon supplementation with high concentration of L-arginine (**Figure 4F**). On the basis of the above results, iNOS inhibitor L-NMMA and Arg-1 inhibitor nor-NOHA were used to explore the potential pathways responsible for exerting immunosuppressive effects in the induced MDSCs. We found that, after addition of iNOS inhibitor, the proliferation of stimulated T cells in the control group was reversed, but that in the TGF-β + GM-CSF–induced MDSCs group remained in a suppressed statement (**Figure 4G**). However, the proliferation of T cells was significantly reversed after the addition of high concentrations of arginase inhibitor to the TGF-β + GM-CSF–induced MDSCs group (**Figure 4H**). Therefore, it was concluded that the immunosuppressive functions of MDSCs induced by TGF-β + GM-CSF were possibly mediated through the Arg-1 pathway but not iNOS or other factors.

### TGF-β + GM-CSF–Induced MDSCs Could Effectively Alleviate the Immune Rejection in the Allo-Skin Graft

**Figure 5:**
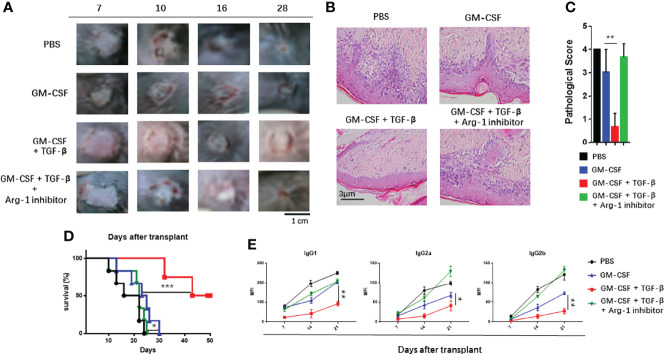
Adoptive transfer of TGF-β + GM-CSF–induced MDSCs could effectively prolong the survival of allo-skin. The recipient female B6 mice were injected intravenously (i.v.) with induced MDSCs (5 × 10^6^) on day 1 before operation and day 7 after operation. The mice in the control group were injected i.v. with vehicle PBS. In addition, TGF-β + GM-CSF–induced MDSCs and Arg-1 inhibitor, nor-NOHA, was administered to another group. **(A)** The photographs of the graft rejection have been shown in the four different groups. **(B)** Representative HE staining of skin grafts on day 21 has been shown (400×). **(C)** Quantification of H&E staining corresponding to the four groups on day 21. **(D)** The survival time of the graft after transplantation have been summarized (n = 6). P values were determined by log-rank tests. *P < 0.05, **P < 0.01, and ***P < 0.001 compared between the different groups. **(E)** The levels of the donor-specific antibodies (DSAs), IgG1, IgG2a, and IgG2b were detected by FCM analysis. The data have been shown as mean ± SEM (n = 3) and were analyzed by one-way ANOVA analysis. *P < 0.05, **P < 0.01, and ***P < 0.001 compared between the different groups.

The induced MDSCs were transferred to the skin of transplant mice to explore their possible ability to suppress immune rejection *in vivo*. It was noted that, as compared with the control groups, adoptive transfer of TGF-β + GM-CSF–induced MDSCs could significantly reduce the graft injury and inflammatory cell infiltration in the graft that was used pathological score to quantize the HE staining of allo-skin in four groups (**Figures 5A–C**) and to prolong the survival time of graft (**Figure 5D**). In addition, the results of the donor-specific anti-antibody (DSA) test indicated that transplant mice had significantly lower levels of Abs in the peripheral blood in the TGF-β + GM-CSF–induced MDSCs treatment group (**Figure 5E**). Another group (comparison group) of transplanted mice was injected intravenously with TGF-β + GM-CSF–induced MDSC cells and then intraperitoneally injected with nor-NOHA (40 mg/kg) for 3 days. It was observed that the survival of the allo-skin in this group was substantially reduced with no significant difference as compared with the PBS treatment group. This finding suggested that MDSCs induced by TGF-β and GM-CSF might exert a marked immunosuppressive effect mainly through Arg-1 pathway in the transplant mice.

### TGF-β + GM-CSF–Induced MDSCs Promoted the Expansion of MDSCs and Tregs *In Vivo*

**Figure 6:**
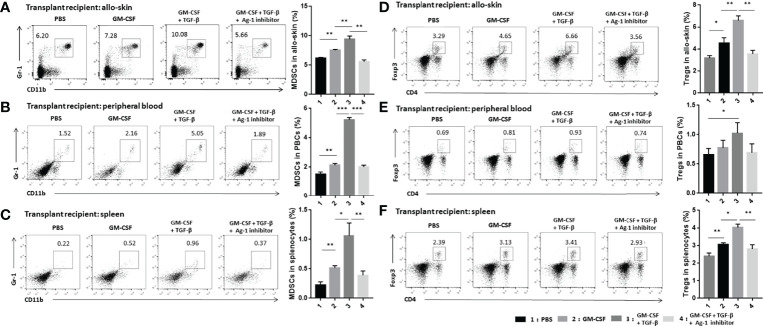
Adoptive transfer of TGF-β + GM-CSF–induced MDSCs could significantly promote the expansion of MDSCs and Tregs in the transplant recipients. Allo-skin mice were divided into four different groups and then treated with PBS, GM-CSF–induced MDSCs, TGF-β + GM-CSF–induced MDSCs, and TGF-β + GM-CSF–induced MDSCs with Arg-1 inhibitor, respectively. Thereafter, allografts, peripheral blood, and spleen of transplant recipients were obtained on day 21 after operation for FCM assay. Typical examples of FCM analysis and proportion of CD11b^+^Gr-1^+^ MDSCs in allografts **(A)**, peripheral blood **(B)**, and spleen **(C)** of the transplanted mice. Typical examples of flow cytometry analysis and percentages of CD4^+^Foxp3^+^ Tregs in allografts **(D)**, peripheral blood **(E)**, and spleen **(F)** of the transplant mice. The data have been shown as mean ± SEM (n = 3) and were analyzed by one-way ANOVA analysis. *P < 0.05, **P < 0.01, and ***P < 0.001 compared between the different groups.

We detected CD11b^+^Gr-1^+^ MDSCs and CD4^+^Foxp3^+^ Tregs in the grafts, peripheral blood, and the spleens of the skin transplant mice on day 21 after the operation. The results indicated that the population of MDSCs in the transplant mice increased significantly after adoptive transferring with TGF-β + GM-CSF–induced MDSCs. The population of MDSCs in the peripheral blood increased the most significantly as compared with other three derived samples (**Figures 6A–C**). In addition, the proportion of Tregs in transplant mice in the TGF-β + GM-CSF–induced MDSCs treatment group was observed to be the highest as compared with other groups (**Figures 6D–F**). This suggested that TGF-β + GM-CSF–induced MDSCs could effectively mobilize more MDSCs and Tregs in the allografted recipient. It is therefore possible that the two types of cells might work together to promote immune tolerance response in the transplant.

## Discussion

MDSCs are a group of cells that can exert negative regulatory effects on the immune response. A large number of previous studies have confirmed that MDSCs are involved in the pathological processes of several immune-related diseases, such as tumors, infections, and autoimmune diseases (19, 23). At present, many studies have highlighted that MDSCs can play a pivotal role in alleviating transplant immune rejection, thereby promoting the development of immune tolerance. It is possible for MDSCs like other regulatory immune cells, such as Tregs, regulatory dendritic cells (DCregs), and regulatory macrophages (Mregs), to effectively suppress the transplant rejection and prolong the graft survival by using cell therapy in the clinic (24–26). A simple and efficient inductive protocol to induce functional MDSCs *in vitro* remains the key to this treatment modality. It has been reported that GM-CSF, which is abundantly present in the tumor microenvironment, is an essential cytokine that can induce MDSCs to exert a negative influence on immune response. TGF-β is a multifunctional cytokine that can participate in modulating the proliferation, differentiation, activation, and migration of various cells, such as hematopoietic cells, stromal cells, and epithelial cells (27). In addition, TGF-β also plays a pivotal role in induction of a robust immune tolerance. This cytokine can also hinder activation and proliferation of T cells directly (27). In addition, Song et al. found that addition of TGF-β to the inductive system of DCs could facilitate DCs to obtain the capacity to promote immune tolerance (28). In the present study, after addition of TGF-β to the system based on GM-CSF for inducing MDSCs, it was found that TGF-β + GM-CSF could significantly induce bone marrow cells to effectively differentiate into more CD11b^+^Gr-1^+^ MDSCs. Several markers on M-MDSCs, such as CD11b, Gr-1, Ly6C, and CD115, were expressed in the GMCSF + TGF-β–induced cells at high level as indicated by flow cytometry assays (29–31), and there were more mononuclear cells than polymorphonuclear cells in the induction system as indicated by Wright–Giemsa staining. Therefore, most of GMCSF + TGF-β–induced MDSCs were M-MDSCs. The expression levels of F4/80, CD11c, and I-Ab were decreased, which potentially suggested that the induced cells were different from macrophages and antigen-presenting cells (APCs). Moreover, upon further analysis of the cell phenotype, we found that TGF-β + GM-CSF–induced MDSCs had significantly higher expression levels of different markers that can negatively regulate immune functions, such as CD274 and CD80. CD274, also known as programmed cell death 1 ligand 1 (PD-L1), can bind to PD-1 on the surface of T cells to prevent the activation and proliferation of T cells (5). CD80 is a costimulatory signal molecule that can combine with cytotoxic T lymphocyte antigen 4 (CTLA-4) on the surface of T cells to exert diverse immunomodulatory effects (32). It has been reported that CD31 can act as a characteristic marker of MDSCs associated with immunosuppression (33). In the present study, the expression of CD31 on the MDSCs induced by TGF-β and GM-CSF decreased substantially. However, the proportions of induced cells with CD31 in the two different groups were very low, which might not have a significant impact on the immunosuppressive ability of the induced cells.

It has been established that the mouse MDSCs can be defined as a population of CD11b^+^Gr-1^+^ cells, which can be further divided into CD11b^+^Ly6C^+^Ly6G^−^ M-MDSCs and CD11b^+^Ly6G^+^Ly6C^−^ G-MDSCs. The two subgroups of the cells differ primarily in expansion conditions and functions. G-MDSCs are mainly expanded in tumors, infection, and autoimmune diseases. In the tumor microenvironment, 70%–80% of all MDSCs have been reported to result in G-MDSCs (34–36). On the other hand, M-MDSCs can serve as the main population of bone marrow–induced MDSCs *in vitro*. In addition, it has been reported that TGF-β can serve as a potential positive factor to improve the expansion and activation of M-MDSCs (7, 18, 19). This was consistent with our research findings in this study. However, as for inhibitory effector molecules in the two subgroups, G-MDSCs can upregulate the expression of reactive oxygen species (ROS) and downregulate that of iNOS. On the contrary, M-MDSCs had higher expression level of iNOS and display lower ROS expression. However, both of the subpopulations could exert potential immunosuppressive function *via* modulation of Arg-1 pathway (37). Although MDSCs have been confirmed to be involved in the regulation of different kinds of immune cells, including T cells, natural killer cells, B cells, macrophages, and dendritic cells, their immunosuppressive effects have been found to be predominantly targeted toward T cells (38). It has been reported that arginase mainly consumes L-arginine in the microenvironment of T cells, which can cause T cells to substantially downregulate the expression of the Cd 3ζ chain due to the lack of this non-essential amino acid. Moreover, the production of cell cycle regulatory proteins in T cells, such as cyclin D3 and CDK4, may also be hindered. Under these circumstances, the proliferation of T cells has been observed to be inhibited (39, 40). Our work showed that after addition of TGF-β, the expression level of Arg-1 was noted to increase significantly. We thus postulated that MDSCs induced by TGF-β in combination with GM-CSF primarily exerted their immunosuppressive effects through affecting the Arg-1 pathway.

It has been found that various types of T cells have immunomodulatory capabilities, including CD4^+^ T cells, CD8^+^ T cells, CD4^−^CD8^−^ T cells, NK T cells, and γδ T cells. Among them, CD4^+^ regulatory T cells, commonly known as Tregs, display an upregulated expression of the Foxp3, which not only serves potentially as a characteristic marker of Tregs but also is essential for maintaining their immune regulatory functions (41). Tregs are currently the most widely studied cell type(s) in the field of lymphocyte-induced immune tolerance. They can effectively inhibit the expansion and activation of various immune cells through altering the levels of various immune regulatory molecules, such as CTLA4, IL-10, IL-4, and TGF-β (42). A number of studies have illustrated that MDSCs can facilitate the expansion of Tregs. There are two different pathways reported for the development of Tregs. The first one has been related to the development of natural Tregs (nTregs), which can be directly differentiated from the thymus. The other one is the one associated with inducible Tregs (iTregs) that can be differentiated from the peripheral antigen–stimulated CD4^+^ T cells. iTregs exert a more powerful role in immune regulation than nTregs (43, 44). For instance, it has been found that the number of Tregs increased significantly and TGF-β + GM-CSF–induced MDSCs can promote their expansion both under *in vitro* and *in vivo* conditions. Several studies also found that *in vitro*-induced MDSCs could promote expansion of Tregs not only in skin graft but also in other organ transplantation, such as corneal, islets, kidney, and heart transplantation (45). In the present study, we found the adoptive transfer of GM-CSF + TGF-β–induced MDSCs could promote expansion of MDSCs in recipient mice. It was reported that sepsis-induced MDSCs adoptively transferred into corneal transplantation mice, resulting in substantial expansion of MDSCs in the recipient bone marrow (46). In addition, a previous study found that *ex vivo*–induced MDSCs transferred into intestinal inflammation mice to enhance proliferation of Tregs. The Tregs produced TGF-β to promote expansion of MDSCs *in vivo* (47). The above research might explain expansion of MDSCs and Tregs in recipient mice of our study. However, an explicit mechanism needs further exploration. Allo-skin transplantation cause severe rejection, resulting in necrosis of grafts within 8–10 days. In the present study, male B6 tail skin was grafted on the dorsal part of the female B6 recipients, which could result in the presence of minor histocompatibility antigen, leading to mild rejection. We used the “minor mismatch” grafted model to better verify the immunomodulatory function of TGF-β–induced MDSC. However, allo-graft transplantation is more common clinically. We will use an suitable allogeneic transplantation model to assess function of TGF-β–induced MDSCs in further study.

In summary, TGF-β in combination with GM-CSF could induce substantially greater number of MDSCs with powerful immunosuppressive function *in vitro*. The TGF-β + GM-CSF–induced MDSCs primarily exerted their immunosuppressive effects *via* regulation of Arg-1 pathway. Moreover, they possessed capability to promote significant expansion of Tregs to participate in establishing a robust immune tolerance. Thus, an effective treatment of transplant immune rejection through cell therapy with the TGF-β + GM-CSF–induced MDSCs can form the basis of novel clinical strategy.

## Data Availability Statement

The original contributions presented in the study are included in the article/**Supplementary Material**. Further inquiries can be directed to the corresponding authors.

## Ethics Statement

The animal study was reviewed and approved by Ethics Committee of Beijing Chaoyang Hospital (Beijing, China).

## Author Contributions

WW_1_,WW_2_, YZ, and PC participated in the research design. PC, ZS, and FZ participated in experimental work of the study and writing of the paper. YZ, JZ, and BY participated in the performance of the research and proposed constructive revisions to the paper. WW, JZ, and XZ participated in the writing of the paper and revising the manuscript. All authors contributed to the article and approved the submitted version.

## Funding

This study was supported by National Natural Science Foundation of China (82070764) and National Natural Science foundation of China (81771720).

## Conflict of Interest

The authors declare that the research was conducted in the absence of any commercial or financial relationships that could be construed as a potential conflict of interest.

## Publisher’s Note

All claims expressed in this article are solely those of the authors and do not necessarily represent those of their affiliated organizations, or those of the publisher, the editors and the reviewers. Any product that may be evaluated in this article, or claim that may be made by its manufacturer, is not guaranteed or endorsed by the publisher.

## Supplementary Material

Supplementary Figure 1 Precursor cells from the C57BL/6J mouse bone marrow cells were cultured in complete medium (CM) as described in Materials and Methods for 4 days with TGF-β or without adding any induced factors to the system. **(A)** The typical dot plot of CD11b^+^Gr-1^+^ cells have been shown by FCM analysis. **(B)** The proportions and numbers of CD11b^+^Gr-1^+^ cells that were counted on the 4^th^ day of cell culture. The data collected from three independent experiments have been shown as mean ± SEM (n = 3) and were analyzed by an unpaired two-tailed Student’s t-test. **P <* 0.05, ***P <* 0.01, ****P <* 0.001 compared between the different groups.

Supplementary Figure 2 Microscopic morphology of the induced MDSCs. Wright–Giemsa staining of MDSCs induced by GM-CSF alone and GM-CSF + TGF-β. The right arrows indicate M-MDSCs, and the green arrows indicate G-MDSCs. The scale bars at the bottom left of images indicate 100 μm.

Supplementary Figure 3 Exploring the source of the Tregs in the T cells and TGF-β + GM-CSF–induced MDSCs co-culture system. **(A)** Typical Dot Plots of CD4^+^Foxp3^+^ Tregs conducted by FCM analysis (the 1^st^ group: the T cells were collected for FCM analysis on the 0 day of co-culture; the 2^nd^ group: the T cells treated by CD25 antibodies were collected for FCM analysis on the day 0 of co-culture; the 3^rd^ group: T cells treated by CD25 antibodies co-culturing with TGF-β + GM-CSF–induced MDSCs for 4 days and then were collected for FCM analysis.). **(B, C)** The percentages and numbers of Tregs in three different group have been summarized. The data have been shown as mean ± SEM (n = 3), which were collected from three independent experiments. **P <* 0.05, ***P <* 0.01, ****P <* 0.001 compared between the different groups.
