# Supplementary figures and images for: TGF-β Enhances Immunosuppression of Myeloid-Derived Suppressor Cells to Induce Transplant Immune Tolerance Through Affecting Arg-1 Expression
Source: Front Immunol. 2022 Jul 7;13:919674. doi: 10.3389/fimmu.2022.919674 (PMC9300822; doi:10.3389/fimmu.2022.919674)

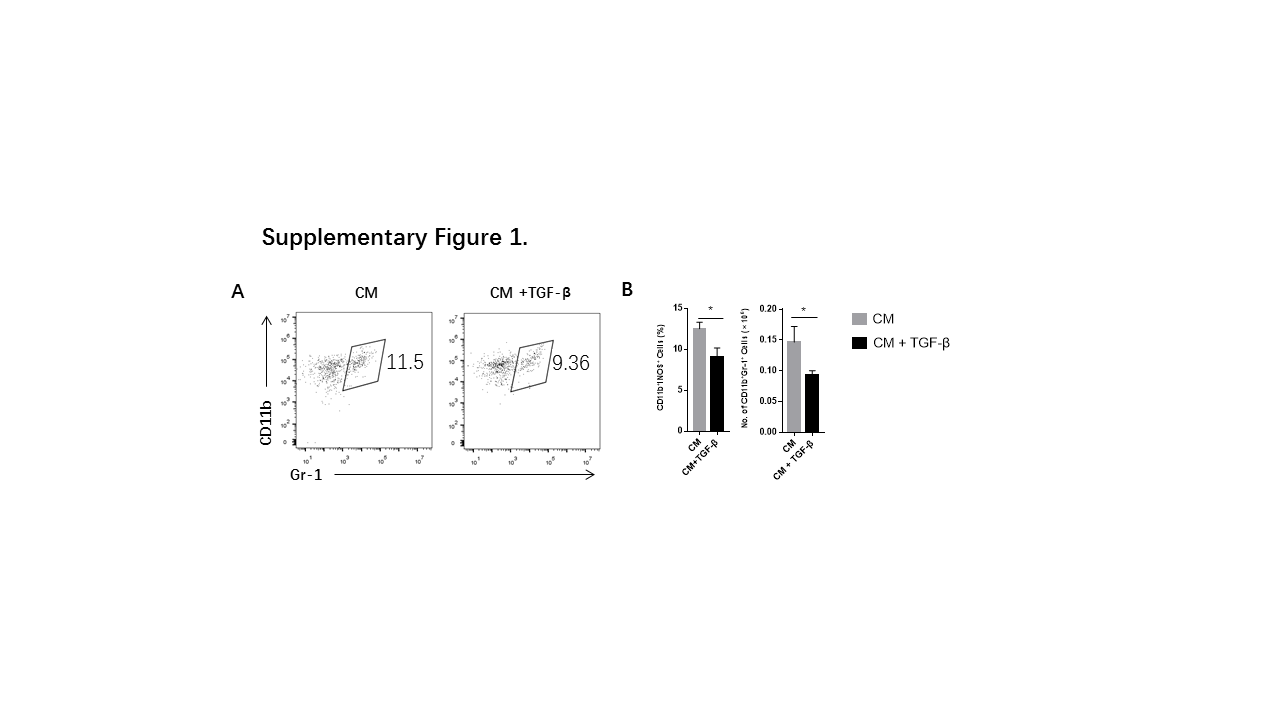

Supplement: Supplementary Figure 1 — Precursor cells from the C57BL/6J mouse bone marrow cells were cultured in complete medium (CM) as described in Materials and Methods for 4 days with TGF-β or without adding any induced factors to the system. (A) The typical dot plot of CD11b+Gr-1+ cells have been shown by FCM analysis. (B) The proportions and numbers of CD11b+Gr-1+ cells that were counted on the 4th day of cell culture. The data collected from three independent experiments have been shown as mean ± SEM (n = 3) and were analyzed by an unpaired two-tailed Student’s t-test. *P < 0.05, **P < 0.01, ***P < 0.001 compared between the different groups. [file Image_1.tif]

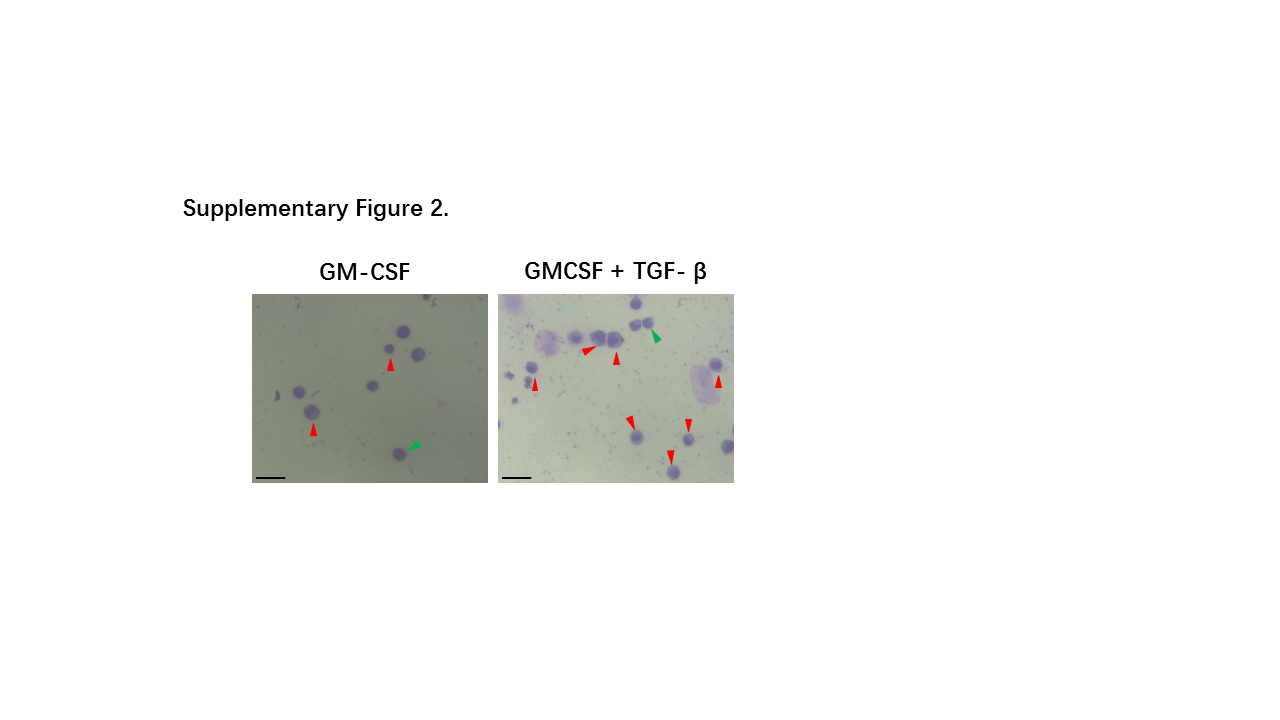

Supplement: Supplementary Figure 2 — Microscopic morphology of the induced MDSCs. Wright–Giemsa staining of MDSCs induced by GM-CSF alone and GM-CSF + TGF-β. The right arrows indicate M-MDSCs, and the green arrows indicate G-MDSCs. The scale bars at the bottom left of images indicate 100 μm. [file Image_2.tif]

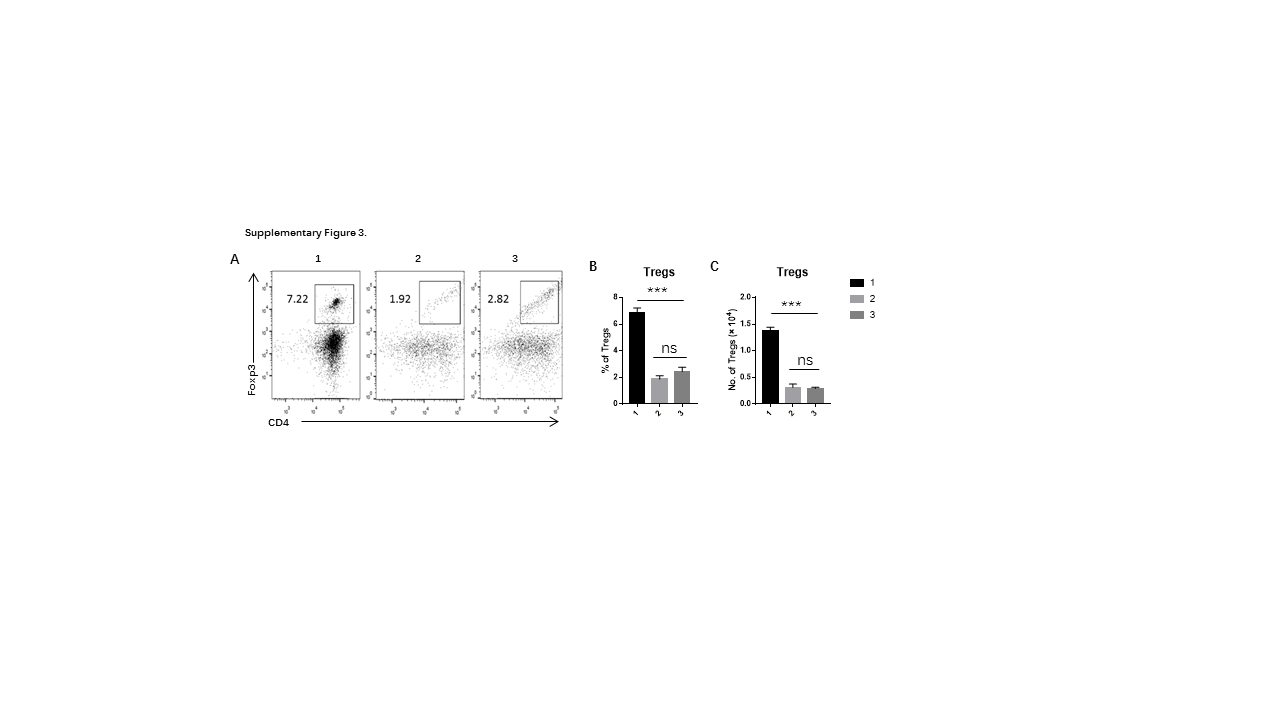

Supplement: Supplementary Figure 3 — Exploring the source of the Tregs in the T cells and TGF-β + GM-CSF–induced MDSCs co-culture system. (A) Typical Dot Plots of CD4+Foxp3+ Tregs conducted by FCM analysis (the 1st group: the T cells were collected for FCM analysis on the 0 day of co-culture; the 2nd group: the T cells treated by CD25 antibodies were collected for FCM analysis on the day 0 of co-culture; the 3rd group: T cells treated by CD25 antibodies co-culturing with TGF-β + GM-CSF–induced MDSCs for 4 days and then were collected for FCM analysis.). (B, C) The percentages and numbers of Tregs in three different group have been summarized. The data have been shown as mean ± SEM (n = 3), which were collected from three independent experiments. *P < 0.05, **P < 0.01, ***P < 0.001 compared between the different groups. [file Image_3.tif]
