# Supplementary material for: TGF-β Enhances Immunosuppression of Myeloid-Derived Suppressor Cells to Induce Transplant Immune Tolerance Through Affecting Arg-1 Expression
Source: Front Immunol. 2022 Jul 7;13:919674. doi: 10.3389/fimmu.2022.919674 (PMC9300822; doi:10.3389/fimmu.2022.919674)
Supplement: Supplementary file 4 [file Table_1.docx]

**Supplementary Table 1** Primer sequences of genes used for Real-time qPCR assay

| **Genes** |  | **Primer sequence (5'to3')** |
| --- | --- | --- |
| HPRT | Forward: | AGTACAGCCCCAAAATGGTTAAG |
|  | Reverse: | CTTAGGCTTTGTATTTGGCTTTTC |
| iNOS | Forward: | CACCAAGCTGAACTTGAGCG |
|  | Reverse: | CGTGGCTTTGGGCTCCTC |
| Arginase-1 | Forward: | CCAGAAGAATGGAAGAGTCAGTGT |
|  | Reverse: | GCAGATATGCAGGGAGTCACC |
| IDO1 | Forward: | TGGCGTATGTGTGGAACCG |
|  | Reverse: | CTCGCAGTAGGGAACAGCAA |
| HO-1 | Forward: | AGGTACACATCCAAGCCGAGA |
|  | Reverse: | CATCACCAGCTTAAAGCCTTCT |
| COX2 | Forward: | CCTTCTCCAACCTCTCCTACT |
|  | Reverse: | ACCTTTTCCAGCACTTCTTTTG |
| NOX2 | Forward: | AGTGCGTGTTGCTCGACAA |
|  | Reverse: | GCGGTGTGCAGTGCTATCAT |
| TGF-β | Forward: | CCACCTGCAAGACCATCGAC |
|  | Reverse: | CTGGCGAGCCTTAGTTTGGAC |
| IL-10 | Forward: | AGCCGGGAAGACAATAACTG |
|  | Reverse: | GGAGTCGGTTAGCAGTATGTTG |
